# A Novel Analytical Method for Trace Ammonium in Freshwater and Seawater Using 4-Methoxyphthalaldehyde as Fluorescent Reagent

**DOI:** 10.1155/2015/387207

**Published:** 2015-09-01

**Authors:** Ying Liang, Yingming Pan, Qing Guo, Hongzhi Hu, Chancui Wu, Qian Zhang

**Affiliations:** ^1^School of Life and Environmental Sciences, Guilin University of Electronic Technology, Guilin 541004, China; ^2^Key Laboratory for the Chemistry and Molecular Engineering of Medicinal Resources, School of Chemistry and Phamaceutical Sciences, Guangxi Normal University, Ministry of Education, Guilin 541004, China

## Abstract

A novel fluorescent reagent for determination of ammonium, 4-methoxyphthalaldehyde (MOPA), was successfully synthesized in this study. Under alkaline conditions, MOPA could reacted with ammonium rapidly at room temperature, producing fluorescent substance which had maximum excitation at 370 nm and emission wavelength at 454 nm. Based on this, a novel fluorescence analysis method was established for the determination of trace ammonium in natural water. Experimental parameters including reagent concentration, pH, reaction equilibrium time, and metal ions masking agent were optimized. The results showed that the optimized MOPA concentration was 0.12 g/L, pH was in the range of 11.2–12.0, and sulfite concentration was 0.051 g/L, respectively. Metal ions masking agent had no obvious effect on the fluorescence signal. With the reaction time of 15 minutes, linear range of this method was between 0.025 and 0.300 *μ*mol/L, and the method detecting limit was 0.0058 *μ*mol/L. The matrix recovery of the proposed method was in the range of 93.6–108.1%. Compared with the OPA method, this method was much more sensitive and rapid without the interference of background peak and would be more suitable for developing a portable fluorescence detection system.

## 1. Introduction

Ammonium is one of the important nitrogen sources of phytoplankton growth [1]. The concentration of ammonium is usually more than micromolar level in mostly continental water and coastal seawater, even up to millimolar level due to environmental pollution [2–4]. However, it is less than micromolar level, even down to nanomolar level in ocean water and some unpolluted freshwater [5, 6]. The accurate measurement of trace ammonium natural water is essential to understand the biogeochemical cycle of nitrogen.

The indophenol blue method (IPB) and* o*-phthalaldehyde (OPA) fluorometric method were the main methods for the determination of ammonium in natural water [7–15]. The indophenol blue method is based on the Berthelot reaction. In the catalysis of nitroprusside, ammonium reacts with hypochlorite and phenol forming indophenol blue, which has a maximum absorbance of 640 nm. The IPB method is used as the standard method to determine ammonium in water by the U.S. Environmental Protection Agency through coupling with a gas-segmented and continuous flow technique [7]. The technique has low sensitivity (LOD 0.6 *μ*mol/L). To meet the determination of trace ammonium in ocean water and some unpolluted freshwater, the sensitivity of the IPB method was obviously improved using a long-path liquid waveguide capillary cell (LWCC) in the later reported work [8]. However, many accompanying weaknesses such as complicated operations and a high reagent blank appeared. The application of the IPB method is still limited in ocean water. OPA method is based on the fluorometric reaction that ammonium reacts with* o*-phthaldialdehyde and sulfite producing highly fluorescent isoindole derivatives, which has the maximum excitation wavelength (*λ*_ex_) at 361–365 nm and the maximum emission wavelength (*λ*_em_) at 422–425 nm. The reaction was firstly reported by Roth in 1971 [9] and was modified by replacing mercaptoethanol with sulfite to provide an OPA method for determination of ammonium in water in 1989 [10]. Afterwards, the OPA method was modified to improve the sensitivity and operability by much work [11–15]. The lower limit of quantitation (1.67 nmol/L) of the OPA method was gained by coupling with flow analysis and solid phase extraction [15], and the method was applied on board to analyze seawater samples collected from 65 stations in the South China Sea, and a detailed ammonium profile in the SEATS station was obtained. However, UV LED (360 nm) had to be used as the excitation light source for conforming to the excitation wavelength of the OPA method, and heating device was also needed in the determination system to provide higher reaction temperature (75°C). UV LED source has many defaults, such as lower light intensity, higher price, and complicated technology [16]. UV LED source and heating device are not very suitable for developing a portable fluorescence detection system. In other words, determination of ammonium is still difficult in field.

If a new fluorescent reagent is prepared for ammonium determination and it can rapidly react with ammonium at room temperature, the determination of ammonium should become much simpler in field. In this paper, a methoxy group, an electron-donating group, was joined to benzene ring of OPA molecule, producing a new fluorescent reagent, 4-methoxyphthalaldehyde (MOPA). And then a novel analytical method was developed for trace ammonium in freshwater and seawater using MOPA as fluorescent reagent.

## 2. Experimental

### 2.1. Reagents and Solutions

MOPA was synthesized in our laboratory. The synthetic method would be introduced in Section 2.2. All of the other chemicals used for analysis were of guaranteed reagent grade, supplied by Aladdin Chemical Reagent Co., China, unless stated otherwise. All solutions were prepared in ultrapure water (resistivity ≥ 18.2 MΩ·cm at 25°C). In detail, 7.8 g/L MOPA solution was made by dissolving 1.95 g of MOPA in 200 mL of methanol (HPLC grade) and diluting to 250 mL with ultrapure water; 1.26 g/L Na_2_SO_3_ solution was made by dissolving 0.63 g of Na_2_SO_3_ in 500 mL ultrapure water and adding 0.20 mL 37% formaldehyde to prevent the solution from being oxidized; 2.0 g/L NaOH solution was prepared by dissolving 1.0 g NaOH in 500 mL of ultrapure water; 40 g/L NaOH solution was prepared by dissolving 20.0 g NaOH in 500 mL of ultrapure water; 300 g/L sodium citrate solution was made by dissolving 30.0 g sodium citrate in 100 mL of ultrapure water; 15 g/L sodium tetraborate buffer solution (*R*_4_) was prepared by dissolving 7.5 g Na_2_B_4_O_7_·10H_2_O in 500 mL ultrapure water; 3.4 g/L OPA solution was made by dissolving 0.34 g of OPA in 20 mL of methanol (HPLC grade) and diluting to 100 mL with ultrapure water; ammonium standard stock solution (1000 mg N/L, 71.4 mmol/L) was purchased from Aladdin Chemical Reagent Co., and the ammonium salt is (NH_4_)_2_SO_4_ in the solution; ammonium standard substock solution (10 mmol/L) was prepared monthly by diluting the stock solution with ultrapure water; the stock and substock solutions were stored at 4°C in a refrigerator while not in use; ammonium working solution (0.1 mmol/L) was prepared daily by diluting 1.0 mL of the substock solution to 100 mL with ultrapure water.

All vessels used in the experiments were firstly soaked with 1 mol·L^−1^ HCl for more than 12 hours, cleaned with reverse osmosis water (resistivity 0.5 MΩ·cm at 25°C), and then soaked with 1 mol·L^−1^ NaOH at least for 12 hours and cleaned thoroughly with ultrapure water before use.

### 2.2. Synthetic Method of MOPA

**Figure 1:**
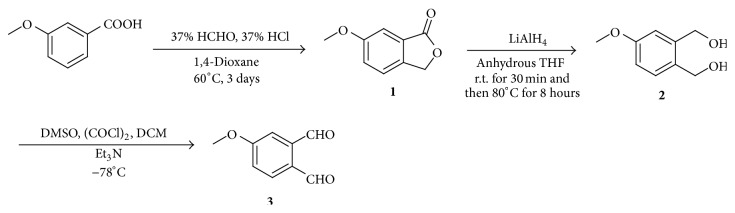
Synthetic route of MOPA.

Synthetic route of MOPA is showed in Figure 1.

*Compound* ***1***. A flask was charged with 15.2 g 3-methoxybenzoic acid and 50 mL dioxane. The mixture was stirred at room temperature and a solution of 50 mL 37% aqueous formalin solution and 50 mL 37% aqueous HCl was added. The reaction was continued at 60°C for 3 days, and then it was cooled at room temperature. The contents of the flask were washed with CH_2_Cl_2_ (dichloromethane/CAS number 75-09-2). The combined organic layer was dried with anhydrous MgSO_4_, filtered, and concentrated under reduced pressure to give crude white solid. This solid was recrystallized from 95% ethyl alcohol to afford 6-methoxyphtalide (compound** 1**) as white needle (11.42 g, 70.1% yield).

*Compound* ***2***. A flask was charged with 11.4 g 6-methoxyphtalide and 80 mL dry tetrahydrofuran (THF), and the mixture was stirred at room temperature. A mixture of 3.4 g LiAlH_4_ and 50 mL dry THF was added slowly when a clear solution was obtained. The reaction was continued at room temperature for 30 minutes, and then it was heated at 80°C for 8 hours. Subsequently, 4 mL H_2_O and 2 mL 15% aqueous NaOH were added to the reaction mixture, respectively. The contents of the flask were stirred at room temperature for 30 minutes. The reaction mixture was filtered and washed with CH_2_Cl_2_. The filtrate was dried with anhydrous MgSO_4_, filtered, and concentrated under reduced pressure to give crude yellow oil (compound** 2**, 8.46 g, 72.2% yield).

*Compound* ***3***. A 250 mL round-bottomed flask equipped with an addition funnel was charged with 6 mL (COCl)_2_ (oxalyl chloride/CAS number 79-37-8) and CH_2_Cl_2_ (60 mL) and the mixture was stirred at −78°C. After the addition of 13.6 mL DMSO (dimethyl sulfoxide/CAS number 67-68-5) and 20 mL CH_2_Cl_2_, the mixture was stirred for 3–5 minutes at −78°C. A solution of compound** 2** (0.06 mol, 10.0 g) and 20 mL CH_2_Cl_2_ and DMSO (*V* :* V* = 10 : 1) was added to the mixture. The mixture was stirred for 30 minutes at −78°C. After the addition of 80 mL Et_3_N, the mixture was stirred for another 10 minutes at −78°C and then the mixture was allowed to warm to room temperature. 200 mL ice water was added to quench the reaction mixture. The contents of the flask were washed with 100 mL CH_2_Cl_2_. The combined organic layer was dried with anhydrous MgSO_4_, filtered, and concentrated* in vacuo*. The residue was purified by silica gel flash column chromatography to give the 4-methoxyphthalaldehyde as yellow needle (5.9 g, 60.1% yield),* mp* 76–78°C. ^1^H NMR (500 MHz, CDCl_3_) *δ* 10.61 (s, 1H), 10.29 (s, 1H), 7.90 (d,* J* = 8.5 Hz, 1H), 7.41 (d,* J* = 2.6 Hz, 1H), 7.19 (dd,* J* = 8.5, 2.6 Hz, 1H), 3.92 (s, 3H); ^13^C NMR (125 MHz, CDCl_3_) *δ* 192.0, 191.0, 163.9, 138.6, 134.7, 129.5, 118.8, 114.8, 56.0. MS(ESI),* m/z*: 165 ([M+H]^+^).

### 2.3. Analytical Procedures of the Proposed Method

10 mL of standard ammonium solution or sample solution with a concentration range of 0.025–0.300 *μ*mol/L was exactly measured into a polypropylene bottle. Appropriate amounts of sodium citrate solution, MOPA solution, Na_2_SO_3_ solution, and NaOH solution were added into the bottle. The concentrations of sodium citrate, OPA, and sodium sulfite in the final solution were 16.8 g/L, 0.12 g/L, and 0.050 g/L, respectively. The pH of the final solution was about 11.4. After all the reagents were added, the mixed solution was tightly sealed and allowed to react for a certain time at room temperature. The fluorescence intensity was measured on a fluorescence spectrophotometer (RF-5301PC, SHIMADZU Co., Ltd., Japan) with excitation wavelength set at 370 nm and emission wavelength at 454 nm. Both the excitation and emission slits of the instrument were set as 5 nm, unless stated otherwise.

## 3. Results and Discussion

### 3.1. Spectral Characteristics of the Reaction Product

#### 3.1.1. Fluorescence Excitation and Emission Spectra

**Figure 2:**
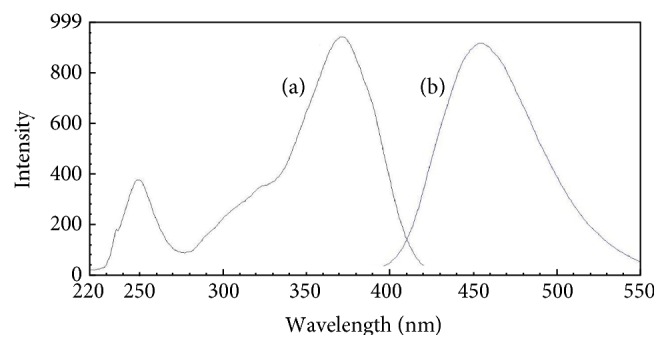
Excitation (a) and emission (b) spectra of the product of ammonium reacting with MOPA.

**Figure 3:**
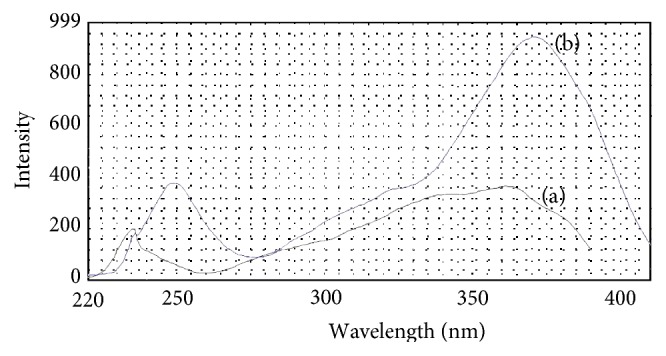
Comparison of excitation spectra. (a) The product of OPA reacting with ammonium; (b) the product of MOPA reacting with ammonium.

**Figure 4:**
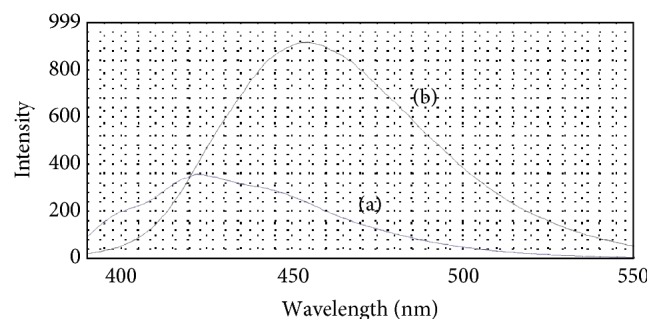
Comparison of emission spectra. (a) The product of OPA reacting with ammonium; (b) the product of MOPA reacting with ammonium.

0.20 *μ*mol/L standard ammonium solution was allowed to react with MOPA and sodium sulfite according to Section 2.3. The excitation and emission spectra of the reaction product are showed in Figure 2. The product had the maximum excitation wavelength (*λ*_ex_) at 370 nm and the maximum emission wavelength (*λ*_em_) at 454 nm. Compared with the product of OPA reacting with ammonium, the maximum excitation and emission wavelength appeared as red shift phenomenon (see Figures 3 and 4), and the maximum excitation and emission wavelength increased 9 nm and 32 nm, respectively. Though the maximum excitation wavelength was located in the nearly UV region, it should be noticed that the fluorescence intensity was still sizable in the wavelength range of 380–410 nm. This means that visible LED at which wavelength ranged from 380 to 410 nm could be chosen as excitation light source to make a portable fluorescence detection system if MOPA is used as fluorescent reagent.

#### 3.1.2. Fluorescence Emission Spectra of Different Solutions

**Figure 5:**
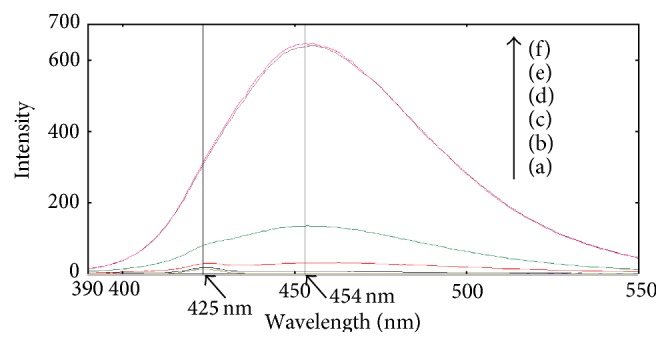
Fluorescence emission spectra of six different solutions. (a) Ultrapure water; (b) NH_4_^+^ + Na_2_SO_3_ + NaOH solution + sodium citrate; (c) MOPA solution; (d) NH_4_^+^ (0.200 *μ*mol/L) + MOPA + Na_2_SO_3_ solution; (e) NH_4_^+^ (0.200 *μ*mol/L) + MOPA + Na_2_SO_3_ + NaOH (pH = 11.4); (f) NH_4_^+^ (0.200 *μ*mol/L) + MOPA + Na_2_SO_3_ + NaOH + sodium citrate (pH = 11.4).

Six solutions were separately prepared and stood for the same time, marked (a), (b), (c), (d), (e), and (f), and their components are listed in the cutline of Figure 5. 30 minutes was set as the standing time but is not necessary, more or less time is also feasible for the experiment in this section. The excitation wavelength was set at 370 nm, and the fluorescence emission spectra of these six solutions were determined, shown in Figure 5. Corresponding to the six solutions, curves in Figure 5 are separately marked (a), (b), (c), (d), (e), and (f) from the bottom up. Curve (a) which has a background peak at 425 nm is the emission spectrum of ultrapure water, and curve (b) almost overlaps curve (a), illuminating that the mixed solution of ammonium, sodium hydroxide, sodium sulfite, and sodium citrate had no fluorescent properties. Curve (c) is the fluorescence emission spectrum of MOPA, showing that MOPA had weak fluorescence. Curve (d) is the fluorescence emission spectrum of the mixed solution of ammonium, MOPA, and sodium sulfite. Compared with curve (c), the intensity of curve (d) had obviously increased, displaying that a fluorescent compound had been produced in solution (d). The difference of solution (d) and solution (e) was pH, and the pH of solution (e) is higher because of addition of sodium hydroxide. The maximum emission wavelength of curve (e) was the same as curve (d), but the fluorescence intensity of curve (e) was much higher than that of curve (d), illuminating that pH was obviously affecting the fluorescence intensity of the solution. Curve (f) almost overlaps curve (e), and the difference of solution (e) and solution (f) lies in the existence of sodium citrate, explaining that sodium citrate had no obvious effect on the fluorescent reaction of the proposed method. The details about the effect of sodium citrate would be discussed in Section 3.2.4. The maximum emission peaks of both curve (e) and curve (f) were located in 454 nm, which appeared as red shift of 29 nm in comparison with curve (a), illuminating that the interference of ultrapure water could be avoided using MOPA as fluorescent reagent. All of above results had confirmed that ammonium reacting with MOPA could produce strong fluorescent compound in the existence of sodium sulfite and sodium hydroxide.

### 3.2. Parameters Optimizing

In this section, effects of parameters such as concentration of MOPA, addition of Na_2_SO_3_ solution, pH in reaction, and concentration of sodium citrate solution on the fluorescence intensity (FI) of blank and 0.200 *μ*mol/L ammonium working solution were investigated based on univariate experimental design.

#### 3.2.1. Effect of MOPA Concentration

**Figure 6:**
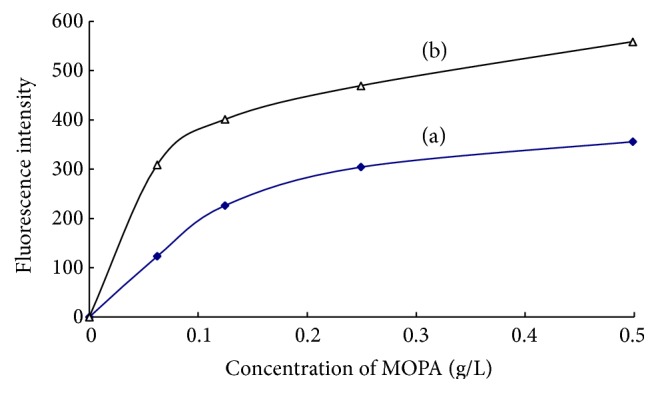
Effect of MOPA concentration on the fluorescence intensities of blank (a) and 0.200 *μ*mol/L ammonium working solution (b).

The concentration of other reagents and experimental conditions were controlled as the described in Section 2.3. The relationship between the fluorescence intensity and the concentration of MOPA in the reaction solution was investigated, shown in Figure 6. The fluorescence intensity of both blank solution and 0.200 *μ*mol/L ammonium working solution increased with an increase in the concentration between 0 and 0.12 g/L and was closed to constant when the MOPA concentration was more than 0.12 g/L. When MOPA was not added in the reaction solution, the fluorescence intensities of both solutions were closed to zero. The results above illuminated that MOPA was an essential fluorescent reagent in the proposed method, and 0.12 g/L of MOPA was enough for the fluorescence reaction. Consequently, the concentration of MOPA in the solution was controlled at 0.12 g/L in the consequent experiment.

#### 3.2.2. Effect of Solution pH

**Figure 7:**
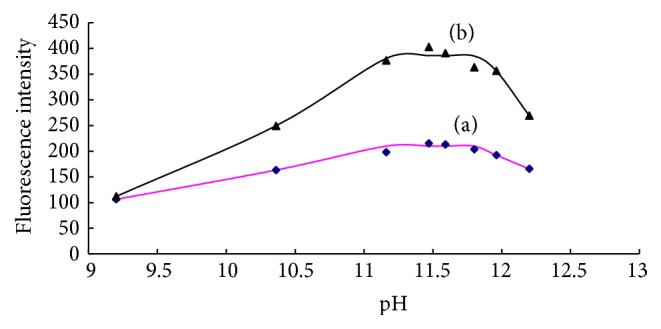
Effect of pH on the fluorescence intensities of blank (a) and 0.200 *μ*mol/L ammonium working solution (b).

Effects of the solution pH on the fluorescence intensity of blank and 0.200 *μ*mol/L ammonium working solution were investigated in the range of 9.2–12.2. The results in Figure 7 illuminated that the fluorescence intensity of both solutions rapidly increased when the pH ranged from 9.2 to 11.2, then closed to a constant in the pH range of 11.2–12.0, and decreased in the final when pH is more than 12.0. It is obvious that the optimum pH range was between 11.2 and 12.0. The pH of the solution was controlled at about 11.4 in the proposed method.

#### 3.2.3. Effect of Na_2_SO_3_ Concentration

**Figure 8:**
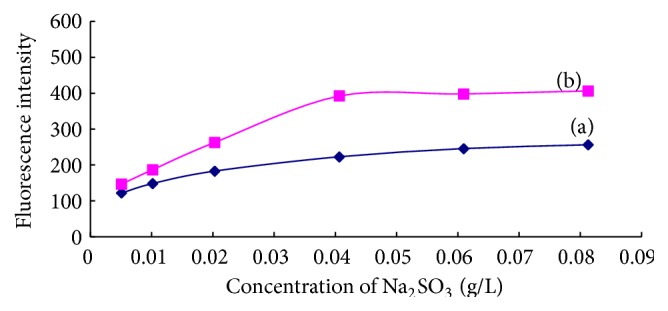
Effect of Na_2_SO_3_ concentration on the fluorescence intensities of blank (a) and 0.200 *μ*mol/L ammonium working solution (b).

The concentration of other reagents and experimental conditions were set as described in Section 2.3. The effect of Na_2_SO_3_ concentration on the fluorescence intensity of blank and 0.200 *μ*mol/L of ammonium working solution was investigated. The results were shown in Figure 8. When the Na_2_SO_3_ concentration in the reaction solution was between 0.041 and 0.081 g/L, the fluorescence intensity of both solutions was closed to maximum constant, illuminating that the amount of Na_2_SO_3_ in the range of 0.041–0.081 g/L was enough for the fluorescent reaction. The concentration of Na_2_SO_3_ was controlled as about 0.051 g/L in the following experiment.

#### 3.2.4. Effect of Sodium Citrate

According to Section 3.2.2, an optimum fluorescence intensity of the reaction solution could be obtained in the pH range of 11.2–12.0. However, precipitation easily occurred in this pH range due to the existence of metal ions in natural water samples. To avoid precipitation of the metal ions, appropriate amount of sodium citrate was added in the reaction solution as metal complexants. Experiments testified that 19 g/L sodium citrate was an optimal choice for most of natural water samples such as seawater, groundwater, and mountain spring water. To investigate the effect of 19 g/L sodium citrate on the fluorescent reaction, different concentration ammonium working solutions in existence and in the absence of sodium citrate were allowed to react with MOPA according to Section 2.3, and the fluorescence intensity signals of these solutions were detected and listed in Table 1. The relationships between fluorescence intensity and concentration of ammonium (*C*_NH_4_^+^_) in existence and in the absence of sodium citrate were FI = 989.3*C*_NH_4_^+^_ + 132.46 (*r* = 0.9974) and FI = 1025*C*_NH_4_^+^_ + 133.06 (*r* = 0.9975), respectively. The intercepts of these two curves were 132.46 and 133.06 and were approximately equal. The slope ratio of these two curves was 0.9652. The results above illustrated that 19 g/L sodium citrate had no obvious effect on the fluorescent reaction.

### 3.3. Reaction Kinetics

**Figure 9:**
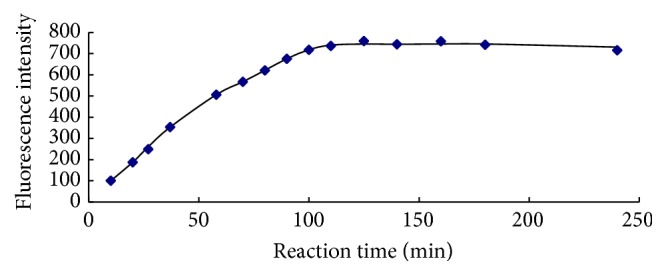
The relationship between fluorescence intensity and reaction time.

0.200 *μ*mol/L ammonium working solution was allowed to react with MOPA in the conditions described in Section 2.3. The FI was detected in different reaction time. To control the FI signal in the range of instrument, the excitation and emission slit were set as 5 nm and 3 nm, respectively. The relationship between FI and reaction time was showed in Figure 9. It was obvious that the FI rapidly increased as the time ranging from 0 to 100 minutes and closed to constant in the range of 100–250 minutes, illuminating that the equilibration time of the reaction was 100 minutes. According to [17], the reaction equilibration time of OPA and ammonium was 180 minutes at room temperature. Consequently, the fluorescent reaction of MOPA and ammonium was much more rapid than that of OPA and ammonium.

### 3.4. Calibration Curves, Sensitivity, Method Detection Limit, and Reproducibility

A typical calibration curve of the proposed method was determined according to Section 2.3. The reaction time was controlled for 15 minutes. The regression equation of the linear curve is FI = 833.4*C*_NH_4_^+^_ + 109.2 (*R* = 0.9946, *n* = 6). The linearity range of the curve was in the range of 0.025–0.300 *μ*mol/L. According to [17], a typical calibration curve of the OPA method was determined at the same reaction time and same temperature as the proposed method. The curve regression equation of the OPA method is FI = 127.2*C*_NH_4_^+^_ + 16.5 (*n* = 5, *R* = 0.9989), and linearity range of the curve was ranged from 0.25 to 1.2 *μ*mol/L. The detailed data of these curves were listed in Table 2. The slope of the proposed method was 6.56 times that of the OPA method, illuminating that the proposed method was much more sensitive than the OPA method.

The reproducibility of the proposed method was evaluated with 4 repetitive determinations of a 0.100 *μ*mol/L ammonium working solution. The relative standard deviation was 2.35%. Four blanks solutions were determined at the same time as the calibration curve, the average FI was 99.581, and the standard deviation was 1.621. The method detection limit, estimated as three times the standard deviations of the blank, was 0.0058 *μ*mol/L. Much lower determination limit should be gained by prolonging the reaction time.

### 3.5. Recovery of the Proposed Method

A surface seawater sample collected from the South China Sea was aged for about one year. Mountain spring water and a groundwater were collected at Yaoshan Scenic Area in Guilin at the day of the experiment. These three samples were separately used as matrix to investigate the recovery of the proposed method. The results were listed in Table 3. The recoveries were in the range of 93.6%–108.1%, showing that both the seawater matrix and freshwater matrix had no interference in ammonium determination. The proposed method was available for seawater and freshwater.

## 4. Conclusion

MOPA was successfully synthesized and could be used as a novel fluorescent reagent for determination of ammonium. Ammonium could rapidly react with MOPA at room temperature producing a strong fluorescent compound, at which maximum excitation and emission wavelength were 370 nm and 454 nm, respectively. Compared with the fluorescent product of ammonium reacting with OPA, the strong fluorescent compound of this proposed reaction appeared as red shift phenomenon, and the excitation emission wavelength was closed to the visible light zone. Based on this, a novel analytical method was proposed for trace ammonium in natural water using MOPA as fluorescent reagent. The proposed method was much more sensitive than the OPA method. The method determination limit was 0.0058 *μ*mol/L when the reaction time was 15 minutes. Much lower determination limit should be gained by prolonging the reaction time. The proposed method is feasible for seawater and freshwater.

## Figures and Tables

**Table 1:** The FI signals of different concentration standard ammonium solution in existence and in the absence of sodium citrate.

Concentration of ammonium (*C*_NH_4_^+^_, *μ*mol/L)	In the absence of sodium citrate	In existence of sodium citrate
0	137.574	138.956
0.100	235.226	227.053
0.200	325.220	319.514
0.300	449.249	437.903

The relationship between FI and *C*_NH_4_^+^_	FI = 1025*C*_NH_4_^+^_ + 133.06 (*r* = 0.9975)	FI = 989.3*C*_NH_4_^+^_ + 132.46 (*r* = 0.9974)

**Table 2:** The data of the calibration curves of the proposed method and the OPA method.

The proposed method		The OPA method	
*C*_NH_4_^+^_, *μ*mol/L	FI signals	C_NH_4_^+^_, *μ*mol/L	FI signals
0	106.558	0	19.495
0.025	122.59	0.2	40.082
0.05	155.229	0.4	67.216
0.1	207.895	0.8	114.717
0.2	262.620	1.2	172.028
0.3	362.817		

The regression equation	FI = 833.4*C*_NH_4_^+^_ + 109.2 (*R* = 0.9946, *n* = 6)	The regression equation	FI = 127.2*C*_NH_4_^+^_ + 16.5 (*R* = 0.9989, *n* = 5)

**Table 3:** The matrix spiked recoveries of the proposed method.

Samples	Spiked concentration of ammonium	Determination results of the samples (*n* = 3)	Recoveries
Seawater	—	0.127 ± 0.007 *μ*moL/L	—
	0.125 *μ*mol/L	0.248 ± 0.010 *μ*moL/L	96.6%
	0.175 *μ*mol/L	0.316 ± 0.017 *μ*moL/L	108.1%

Mountain spring water	—	0.089 ± 0.004 *μ*moL/L	—
	0.125 *μ*mol/L	0.207 ± 0.007 *μ*moL/L	94.4%
	0.175 *μ*mol/L	0.255 ± 0.012 *μ*moL/L	94.9%

Groundwater	—	0.062 ± 0.003 *μ*moL/L	—
	0.125 *μ*mol/L	0.179 ± 0.004 *μ*moL/L	93.6%
	0.175 *μ*mol/L	0.240 ± 0.008 *μ*moL/L	101.7%

## References

[1] Domingues R. B., Barbosa A. B., Sommer U., Galvão H. M. (2011). Ammonium, nitrate and phytoplankton interactions in a freshwater tidal estuarine zone: potential effects of cultural eutrophication. *Aquatic Sciences*.

[2] Hinkle S. R., Böhlke J. K., Duff J. H., Morgan D. S., Weick R. J. (2007). Aquifer-scale controls on the distribution of nitrate and ammonium in ground water near La Pine, Oregon, USA. *Journal of Hydrology*.

[3] Eddy F. B. (2005). Ammonia in estuaries and effects on fish. *Journal of Fish Biology*.

[4] Zhang X.-Q., Xia X.-H., Yang Z.-F. (2007). Reasons of high concentration ammonium in Yellow River, China. *Environmental Science*.

[5] Brzezinski M. A. (1988). Vertical distribution of ammonium in stratified oligotrophic waters. *Limnology and Oceanography*.

[6] Hu H., Liang Y., Li S., Guo Q., Wu C. (2014). A modified *o*-phthalaldehyde fluorometric analytical method for ultratrace ammonium in natural waters using edta-naoh as buffer. *Journal of Analytical Methods in Chemistry*.

[7] US Environmental Protection Agency (1997). *EPA Method 349.0: Determination of Ammonia in Estuarine and Coastal Waters by Gas Segmented Continuous Flow Colorimetric Analysis*.

[8] Li Q. P., Zhang J.-Z., Millero F. J., Hansell D. A. (2005). Continuous colorimetric determination of trace ammonium in seawater with a long-path liquid waveguide capillary cell. *Marine Chemistry*.

[9] Roth M. (1971). Fluorescence reaction for amino acids. *Analytical Chemistry*.

[10] Genfa Z., Dasgupta P. K. (1989). Fluorometric measurement of aqueous ammonium ion in a flow injection system. *Analytical Chemistry*.

[11] Aminot A., Kérouel R., Birot D. (2001). A flow injection-fluorometric method for the determination of ammonium in fresh and saline waters with a view to in situ analyses. *Water Research*.

[12] Amornthammarong N., Zhang J.-Z. (2008). Shipboard fluorometric flow analyzer for high-resolution underway measurement of ammonium in seawater. *Analytical Chemistry*.

[13] Amornthammarong N., Zhang J.-Z., Ortner P. B. (2011). An autonomous batch analyzer for the determination of trace ammonium in natural waters using fluorometric detection. *Analytical Methods*.

[14] Horstkotte B., Duarte C. M., Cerdà V. (2011). A miniature and field-applicable multipumping flow analyzer for ammonium monitoring in seawater with fluorescence detection. *Talanta*.

[15] Zhu Y., Yuan D., Huang Y., Ma J., Feng S. (2013). A sensitive flow-batch system for on board determination of ultra-trace ammonium in seawater: method development and shipboard application. *Analytica Chimica Acta*.

[16] Dobrinsky A., Shatalov M., Gaska R., Shur M. Physics of visible and UV LED devices.

[17] Chen X., Tan Y., Li M. J. (2001). Fluorometric measurement of ammonina in seawater. *Journal of Xiamen University (Natural Science)*.

